# Lymphopenia in patients affected by SARS-CoV-2 infection is caused by margination of lymphocytes in large bowel: an [^18^F]FDG PET/CT study

**DOI:** 10.1007/s00259-022-05801-0

**Published:** 2022-04-29

**Authors:** Alberto Signore, Chiara Lauri, Marzia Colandrea, Marco Di Girolamo, Erika Chiodo, Chiara Maria Grana, Giuseppe Campagna, Antonio Aceti

**Affiliations:** 1 Nuclear Medicine Unit, Department of Medical-Surgical Sciences and of Translational Medicine, Faculty of Medicine and Psychology, Sapienza University of Rome, Rome, Italy; 2 Nuclear Medicine Division, European Institute of Oncology – IRCCS, Milan, Italy; 3 Radiology Unit, AOU Sant’Andrea, Sapienza University of Rome, Rome, Italy; 4 Infection Unit, Department NESMOS, Sapienza University of Rome, Rome, Italy

**Keywords:** COVID-19, Pneumonia, [^18^F]FDG PET/CT, SARS-CoV-2, Lymphopenia

## Abstract

**Background:**

To investigate the cause of lymphopenia in patients with newly diagnosed COVID-19, we measured [^18^F]FDG uptake in several tissues, including the ileum, right colon, and caecum at diagnosis and after recovery and correlated these measurements with haematological parameters.

**Methods:**

We studied, by [^18^F]FDG PET/CT, 18 newly diagnosed patients with COVID-19. Regions of interest were drawn over major organs and in the terminal ileum, caecum, and right colon, where the bowel wall was evaluable. Five patients were re-examined after recovery, and three of them also performed a white blood cell scan with ^99m^Tc-HMPAO-WBC on both occasions. Complete blood count was performed on both occasions, and peripheral blood lymphocyte subsets were measured at diagnosis. Data were analysed by a statistician.

**Results:**

Patients had moderate severity COVID-19 syndrome. Basal [^18^F]FDG PET/CT showed focal lung uptake corresponding to hyperdense areas at CT. We also found high spleen, ileal, caecal, and colonic activity as compared to 18 control subjects. At recovery, hypermetabolic tissues tended to normalize, but activity in the caecum remained higher than in controls. Regression analyses showed an inverse correlation between CD4 + lymphocytes and [^18^F]FDG uptake in the caecum and colon and a direct correlation between CD8 + lymphocytes and [^18^F]FDG uptake in lungs and bone marrow. WBC scans showed the presence of leukocytes in the caecum and colon that disappeared at recovery.

**Conclusions:**

These findings indicate that lymphopenia in COVID-19 patients is associated with large bowel inflammation supporting the hypothesis that CD4 + lymphocytes migrate to peripheral lymphoid tissues in the bowel.

**Supplementary Information:**

The online version contains supplementary material available at 10.1007/s00259-022-05801-0.

## Introduction

Coronavirus disease 2019 (COVID-19) is a severe syndrome that emerged in December 2019 in Wuhan, China, and rapidly spread worldwide, allowing the declaration of a pandemic by the World Health Organization (WHO) in March 2020. Novel coronavirus, called severe acute respiratory syndrome coronavirus 2 (SARS-CoV-2), belongs to a large family of respiratory viruses that can cause a wide spectrum of respiratory diseases and extra-pulmonary manifestations. Clinical presentation is extremely variegated, ranging from asymptomatic cases or pauci-symptomatic cases with fever, dry cough, asthenia, and myalgia to acute respiratory distress syndrome (ARDS) due to bilateral interstitial pneumonia that sometimes may require support in the intensive care unit (ICU).

Cytokine dysregulation plays a major role in the development and progression of these diseases [1]. An uncontrolled pro-inflammatory cytokine and chemokine release known as “cytokine storm” [2] is responsible for an initial over-activation of T-cells, enhanced vascular permeability, disseminated intravascular coagulation, ARDS, and other systemic effects known as cytokine released syndrome (CRS). T-cell depletion is also a common finding, frequently observed since the initial phase of COVID-19. A possible explanation is that an up-regulation of pro-inflammatory cytokines may be responsible for T-lymphocytes exhaustion and their depletion from plasma and the accumulation of CD8 + cells in the lungs where they exert their cytotoxic action, thus causing immune-mediated tissue injury [3, 4]. Nevertheless, the reasons for lymphopenia remain unclear.

To the best of our knowledge, no one has yet investigated the biodistribution of [^18^F]FDG in patients with newly diagnosed COVID-19 aiming at better understanding hypermetabolism of different tissues as a marker of inflammation and correlating the inflammatory status with haematological parameters.

## Methods

### Patients

In March–April 2020, we studied the first two patients affected by COVID-19 syndrome and, based on the quantitative analysis of [^18^F]FDG in tissues, we calculated a sample size of *n* = 18 patients (14 males and 4 females; mean age 60.29 ± 9.47) to find significant differences in spleen uptake of FDG vs. normal subjects.

During the second wave of the COVID-19 pandemic, from November 2020 to June 2021, we performed [^18^F]FDG PET/CT in several patients for clinical purposes. Subsequently, for this study, we retrospectively recruited 14 patients (12 males and 3 females) with newly diagnosed COVID-19 syndrome admitted to the emergency department of Sant’Andrea hospital in Rome (*n* = 11) or in IEO Milan (*n* = 3). Seven of them were re-examined after therapy and a double negative RT-PCR test for SARS-CoV-2 (1.5 to 4 months after diagnosis).

Inclusion criteria for this study were the availability of a [^18^F]FDG PET/CT scan, presence of moderate severity of COVID-19 syndrome with pneumonia, fever, oxygen saturation > 90%, low lymphocyte count, the positivity of nasopharyngeal swab with RT-PCR test for SARS-CoV-2 not more than 3 days before PET.

Exclusion criteria were critical patients that needed ICU and external breathing support during the scan, patients that started immunosuppressive therapies for COVID-19 or for other reasons, chemotherapy, biologic therapy, radiation therapy, pregnancy, or nursing women.

Four more patients (3 males and 1 female) were referred to our Nuclear Medicine Unit by orthopaedic surgeons to perform scintigraphy with ^99m^Tc-HMPAO labelled autologous leukocytes (^99m^Tc-WBC) for suspected infection of knee prosthesis (in three cases) or tibial fracture (in one case). These patients performed early abdominal images at 2 h after administration of ^99m^Tc-WBC and, in one case, also images at 20 h. Three patients developed a fever on the first day after the early scan and were diagnosed with SARS-CoV-2 infection. The other patient developed fever and respiratory symptoms after the 20 h scan and was also diagnosed with SARS-CoV-2 infection. All patients were included in the study because fulfilled the inclusion criteria. One of them performed two [^18^F]FDG PET/CT scans (at diagnosis and after therapy).

Patients were classified as moderately-lymphopenic or severely-lymphopenic according to lymphocyte count higher or lower than 1000 cells/µl, respectively.

Therapy, with personalized doses, included steroids, remdesivir, paracetamol, and hydroxychloroquine.

In summary, we analysed [^18^F]FDG PET/CT images of 18 patients at the time of diagnosis and in 7 patients after recovery (Supplementary Table). The ^99m^Tc-WBC scan was performed in 4 patients at the time of diagnosis and, in 3, after recovery.

At time of each scan, we withdraw 10 ml of blood to perform complete blood count and cytofluorimetric measurement of CD3 + , CD19 + , CD45 + , CD3 + CD4 + /CD45 + , CD3 + CD8 + /CD45 + , and CD16 + CD56 + /CD45 + lymphocyte subsets (only available in 14 patients).

As control group, we selected 18 age matched patients (6 males and 12 females; mean age 58.71 ± 8.92), non-diabetics, non taking metformin, who performed [^18^F]FDG PET/CT mainly for oncological reasons but showed a normal distribution of FDG without any pathological uptake (Supplementary Table).

The standard routine blood test was performed almost every week, and blood counts every 2–3 days. Full recovery, with a double negative nasopharyngeal swab, occurred between 33 and 90 days from diagnosis. Follow-up scans were completed in October 2021.

### [^18^F]FDG PET/CT

Both basal and follow-up [^18^F]FDG PET/CT scans were performed using a hybrid PET/CT system (Biograph Horizon, Siemens, Germany) after receiving a standard dose of [^18^F]FDG of 4–5 MBq/Kg. Patients were checked for blood glycaemia before injection and hydrated with 500 ml saline intravenously. Images were strictly acquired 60 ± 5 min after [^18^F]FDG injection, for 2.5 min per bed position from the pelvis to head. The CT scans were performed with the following parameters: matrix of 512 × 512, slice thickness of 3.75 mm, 170 kVp, 90 mA, 0.8/s tube rotation. After the acquisition, the attenuation corrected PET images were automatically fused with CT images and displayed in maximum intensity projection (MIP) in the axial, coronal and sagittal planes.

CT scans were acquired with a slice thickness of 3.75 mm to minimize mismatches between CT and PET scans. Furthermore, the PET scan started from the pelvic region immediately after CT acquisition (therefore less than a 2 min delay from CT to pelvic PET).

**Fig. 1:**
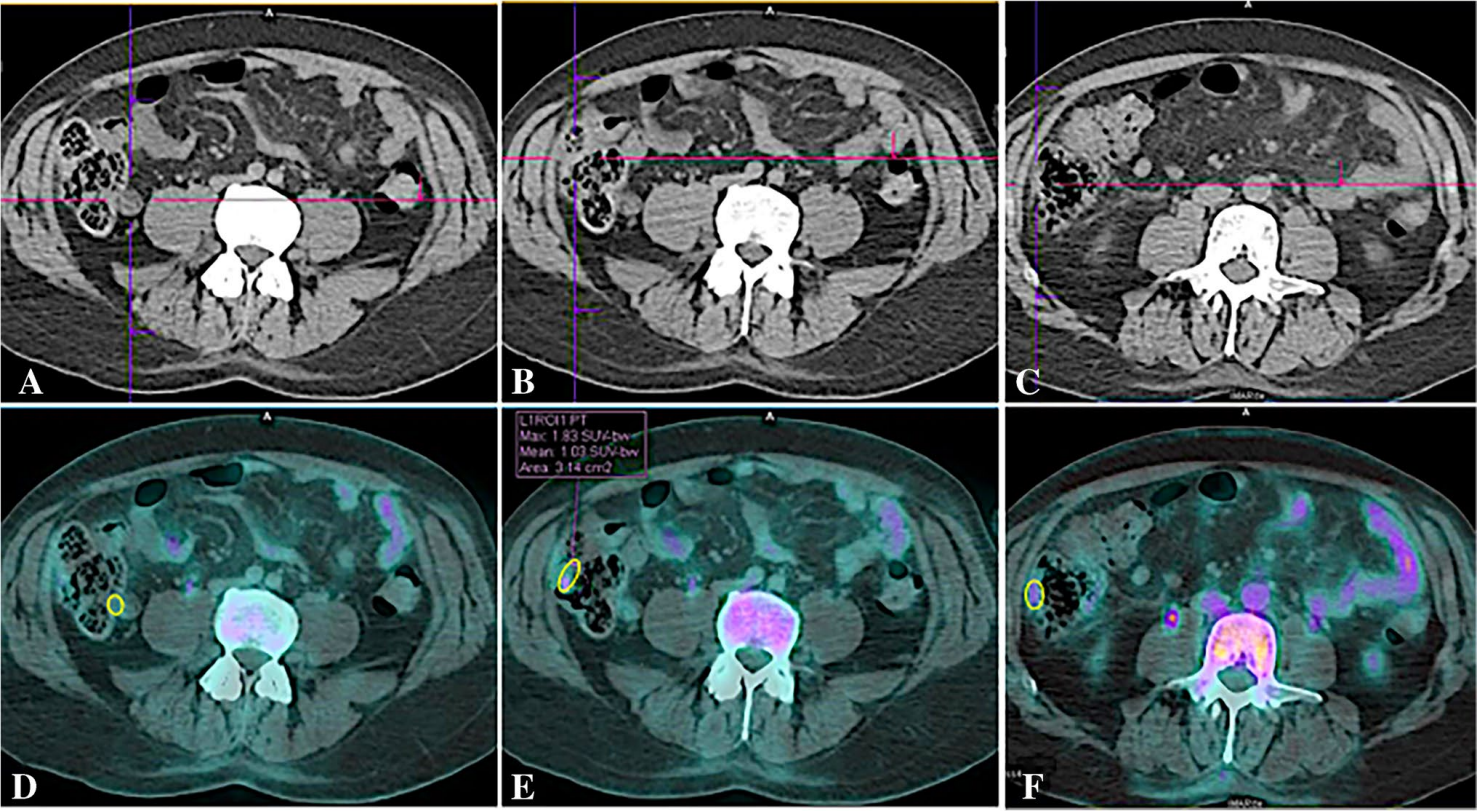
Axial PET/CT images in a patient at the time of diagnosis, showing how we measured the activity in the bowel wall. Regions with adequate thickened walls were selected by the radiologist on CT slices (**A**–**C**), and then circular ROIs were drawn over the corresponding PET/CT images (**D**–**F**). The software provides the measurement of SUV_max_, SUV_mean_, and total ROI area. Images A and D show the ROI in the terminal ileum; B and C the ROI in the caecum, and C and F the ROI in the ascending colon of a newly diagnosed patient

Quantitative image analysis was performed, on axial sections, by measuring the mean standardized uptake value (SUV_mean_) in circular regions of interest (ROIs) drawn in upper, middle, and lower, left and right lung, liver, thoracic aorta, bone marrow (D3, D6 D9), spleen and bowel [5–8]. The intestinal ROIs in the terminal ileum, caecum, and right colon, were drawn on CT scans by an expert radiologist, blind, examining axial and coronal sections. ROIs were drawn in correspondence with a portion of the evaluable thickened intestinal wall, thus allowing to differentiate of the wall from intraluminal faecal material. The same ROIs were transposed to PET images, and SUV_mean_ was calculated (Fig. 1). Three to five ROIs were drawn for each bowel segment.

### ^99m^Tc-HMPAO white blood cell scintigraphy

Ex vivo labelling of WBCs with ^99m^Tc-HMPAO was performed according to European Association of Nuclear Medicine (EANM) procedural Guidelines [9], using the Leukokit® [10]. Labelling efficiency (LE) ranged between 63 and 87.6%. The amount of ^99m^Tc-HMPAO-WBCs per patient was between 600 and 700 MBq.

Early abdominal images for in vivo quality control were acquired, according to EANM guidelines [11, 12], at 2 h post-injection, using a 512 × 512-pixel matrix for 300 s. The 3 and 20 h post-injection images were acquired only of the knees and legs for the suspected osteomyelitis.

### Statistical analysis

The sample size was estimated considering data published by Bai et al. [13]. We determined the pooled standard deviation of SUVmean of spleen between the two groups (Sp = 0.58) and assumed a difference in spleen metabolism *d* = 0.56; therefore, with an *α* = 5% and a power of 80%, we calculated a sample size of *n* = 18 patients per group.

Continuous variables are presented as mean or median ± standard deviation (SD) and 95% Confidence Interval (95%CI). Categorical variables are expressed as absolute frequencies and percentages – *n* (%).

The comparisons in Tables 1, 2, and 3 were performed by Student *t*-test (presence of normality) or Mann–Whitney test (absence of normality). In presence of heteroscedasticity, we used the correction of Satterthwaite. Multiple comparisons in Table 1 were corrected by Benjamini–Hochberg method.

**Table 1:** Blood count values and [^18^F]FDG uptake (SUV_mean_) in different tissues at diagnosis and at recovery in COVID-19 patients compared to control subjects

Parameter	Group A COVID-19 at diagnosis (*n* = 18) Mean ± SD (95%CI) or median (95%CI)	Group B COVID-19 after therapy (*n* = 7) Mean ± SD (95%CI) or median (95%CI)	Group C Control subjects (*n* = 18) Mean ± SD (95%CI) or median (95%CI)	*P* (A vs. C)	*P* (B vs. C)
WBC (cells/µl)	8.00 ± 3.47 (6.27 to 9.72)	6.41 ± 2.47 (3.82 to 9.01)	8.20 ± 2.15 (7.09 to 9.30)	0.84	0.11
Neutrophils (cells/µl)	5.92 (3.77 to 7.26)	3.43 (3.02 to 9.28)	4.96 (4.35 to 6.52)	0.66	0.12
Lymphocytes (cells/µl)	1.05 (0.57 to 1.67)	1.73 (0.47 to 2.52)	1.85 (1.65 to 2.68)	**0.0002**	0.22
Monocytes (cells/µl)	0.68 ± 0.22 (0.57 to 0.78)	0.46 ± 0.16 (0.31 to 0.61)	0.53 ± 0.11 (0.47 to 0.59)	**0.01**	0.24
Left lung (SUVmean)	1.61 ± 0.75 (1.24 to 1.98)	0.56 ± 0.23 (0.35 to 0.77)	0.57 ± 0.16 (0.49 to 0.65)	** < 0.0001**	0.93
Right lung (SUVmean)	1.31 (0.75 to 1.75)	0.45 (0.32 to 0.89)	0.51 (0.42 to 0.55)	** < 0.0001**	0.56
Spleen (SUVmean)	2.34 ± 0.43 (2.12 to 2.55)	1.87 ± 0.25 (1.64 to 2.11)	2.03 ± 0.26 (1.90 to 2.16)	**0.04**	0.19
Liver (SUVmean)	2.62 ± 0.53 (2.35 to 2.88)	2.39 ± 0.37 (2.05 to 2.73)	2.49 ± 0.34 (2.31 to 2.66)	0.53	0.53
Bone marrow (SUVmean)	2.07 (1.52 to 2.60)	1.86 (1.36 to 3.54)	1.78 (1.73 to 1.96)	0.26	0.73
Terminal ileum (SUVmean)	1.24 ± 0.31 (1.09 to 1.39)	1.08 ± 0.33 (0.77 to 1.38)	0.90 ± 0.31 (0.75 to 1.06)	**0.006**	0.24
Caecum (SUVmean)	1.29 ± 0.49 (1.04 to 1.53)	1.16 ± 0.46 (0.74 to 1.58)	0.73 ± 0.21 (0.62 to 0.83)	**0.0004**	0.09
Right colon (SUVmean)	1.21 (0.68 to 1.63)	0.88 (0.76 to 2.12)	0.73 (0.63 to 0.84)	**0.0008**	**0.02**
Thoracic aorta (SUVmean)	2.00 ± 0.48 (1.76 to 2.24)	1.75 ± 0.18 (1.59 to 1.92)	1.94 ± 0.28 (1.80 to 2.09)	0.99	0.16

Data in bold indicate statistically detectable differences

**Table 2:** Blood count values and [^18^F]FDG uptake (SUV_mean_) in different tissues of severely-lymphopenic and moderately-lymphopenic COVID-19 patients

Parameter	Severely-lymphopenic patients (*n* = 9) Mean ± SD (95%CI) or median (95%CI)	Moderately-lymphopenic patients (*n* = 9) Mean ± SD (95%CI) or median (95%CI)	*P*
WBC (cells/µl)	7.97 ± 4.60 (4.43 to 11.50)	8.02 ± 2.11 (6.40 to 9.65)	0.97
Neutrophils (cells/µl)	6.68 ± 4.49 (3.23 to 10.14)	5.72 ± 1.77 (4.36 to 7.08)	0.56
Lymphocytes (cells/µl)	0.57 (0.29 to 0.84)	1.67 (1.25 to 1.72)	**0.0004**
Monocytes (cells/µl)	0.60 ± 0.24 (0.41 to 0.78)	0.76 ± 0.16 (0.64 to 0.88)	0.12
Left lung (SUV_mean_)	1.74 ± 0.70 (1.20 to 2.28)	1.48 ± 0.82 (0.85 to 2.11)	0.48
Right lung (SUV_mean_)	1.29 ± 0.81 (0.66 to 1.91)	1.52 ± 0.79 (0.91 to 2.12)	0.55
Spleen (SUV_mean_)	2.37 ± 0.33 (2.12 to 2.62)	2.30 ± 0.54 (1.89 to 2.72)	0.77
Liver (SUV_mean_)	2.66 ± 0.51 (2.27 to 3.06)	2.57 ± 0.57 (2.13 to 3.01)	0.72
Bone marrow (SUV_mean_)	2.30 ± 0.83 (1.66 to 2.94)	2.33 ± 1.15 (1.44 to 3.22)	0.95
Terminal ileum (SUV_mean_)	1.33 ± 0.29 (1.11 to 1.55)	1.15 ± 0.32 (0.91 to 1.40)	0.24
Caecum (SUV_mean_)	1.59 ± 0.41 (1.27 to 1.91)	0.98 ± 0.35 (0.71 to 1.25)	**0.004**
Right colon (SUV_mean_)	1.60 ± 0.58 (1.15 to 2.05)	0.96 ± 0.64 (0.47 to 1.45)	**0.04**
Thoracic aorta (SUV_mean_)	1.98 (1.66 to 2.26)	1.92 (1.53 to 2.58)	0.79

Data in bold indicate statistically detectable differences

**Table 3:** Lymphocyte subsets in severely-lymphopenic and moderately-lymphopenic COVID-19 patients

Parameter (normal values)	Severely-lymphopenic patients (*n* = 7) Mean ± SD (95%CI) or median (95%CI)	Moderately-lymphopenic patients (*n* = 7) Mean ± SD (95%CI) or median (95%CI)	*P*
% CD3 + (67.0 to 76.0%)	68.05 ± 12.58 (56.42 to 79.68)	72.05 ± 12.58 (56.42 to 79.68)	0.97
Absolute CD3 + (1100 to 1700)	438.71 ± 179.37 (272.82 to 604.60)	948.71 ± 179.37 (272.82 to 604.60)	0.56
% CD3 + CD4 + /CD45 + (36.0 to 46.0%)	28.89 ± 16.36 (21.72 to 56.05)	48.89 ± 16.36 (21.72 to 56.05)	**0.0004**
Absolute CD3 + CD4 + /CD45 + (700 to 1100)	291.43 ± 148.59 (205.64 to 377.22)	691.43 ± 148.59 (205.64 to 377.22)	0.12
% CD3 + CD8 + /CD45 + (31.0 to 40.0%)	25.62 ± 13.82 (11.12 to 40.12)	35.62 ± 13.82 (11.12 to 40.12)	0.48
Absolute CD3 + CD8 + /CD45 + (500 to 900)	119.43 ± 53.77 (88.38 to 150.48)	619.43 ± 53.77 (88.38 to 150.48)	0.55
% CD19 + (11.0 to 16.0%)	13.81 ± 9.19 (6.31 to 23.32)	14.81 ± 9.19 (6.31 to 23.32)	0.77
Absolute CD19 + (200 to 400)	107.14 ± 77.58 (35.39 to 178.90)	207.14 ± 77.58 (35.39 to 178.90)	0.72
% CD16 + CD56 + /CD45 + (10.0 to 19.0%)	15.40 ± 7.82 (8.17 to 22.63)	21.40 ± 7.82 (8.17 to 22.63)	**0.004**
Absolute CD16 + CD56 + /CD45 + (200 to 400)	102.14 ± 69.75 (37.63 to 166.65)	202.14 ± 69.75 (37.63 to 166.65)	**0.04**
CD4 + /CD8 + (1.0 to 1.9)	2.32 ± 2.40 (-0.20 to 4.83)	1.92 ± 2.40 (− 0.20 to 4.83)	0.79

Data in bold indicate statistically detectable differences

In Table 4, the correlations were tested by Pearson (presence of normality) or Spearman (absence of normality) coefficients.

**Table 4:** Correlation between [^18^F]FDG uptake in tissues and lymphocyte subsets in COVID-19 patients at diagnosis

Parameter (SUV_mean_)	All lymphocyte (cells/µl) Correlation coefficient (95%CI) (*n* = 18)	CD3 + /CD4 + /CD45 + (cells/µl) Correlation coefficient (95%CI) (*n* = 14)	CD3 + /CD8 + /CD45 + (cells/µl) Correlation coefficient (95%CI) (*n* = 14)
Left lung	− 0.15 (− 0.58 to 0.34) *p* = 0.55	− 0.40 (− 0.77 to 0.16) *p* = 0.15	**0.81 (0.49 to 0.94)** ***p* = 0.0004**
Right lung	0.22 (− 0.27 to 0.62) *p* = 0.37	− 0.04 (− 0.56 to 0.50) *p* = 0.90	**0.82 (0.51 to 0.94)** ***p* = 0.0003**
Spleen	0.03 (− 0.44 to 0.49) *p* = 0.90	0.03 (− 0.51 to 0.55) *p* = 0.93	0.14 (− 0.42 to 0.63) *p* = 0.62
Liver	0.02 (− 0.45 to 0.49) *p* = 0.92	0.27 (− 0.30 to 0.70) *p* = 0.36	0.36 (− 0.21 to 0.75) *p* = 0.21
Bone marrow	0.04 (− 0.43 to 0.50) *p* = 0.87	0.06 (− 0.49 to 0.57) *p* = 0.84	**0.71 (0.30 to 0.90)** ***p* = 0.004**
Terminal ileum	− 0.21 (− 0.62 to 0.28) *p* = 0.40	− 0.26 (− 0.70 to 0.31) *p* = 0.36	− 0.06 (− 0.57 to 0.48) *p* = 0.83
Caecum	− **0.74 (**− **0.90 to** − **0.42)** ***p* = 0.0004**	− **0.78 (**− **0.93 to** − **0.42)** ***p* = 0.001**	− 0.18 (− 0.65 to 0.38) *p* = 0.53
Right colon	− **0.67 (**− **0.87 to** − **0.30)** *p* = **0.002**	− **0.71 (**− **0.90 to** − **0.30)** ***p* = 0.004**	0.09 (− 0.46 to 0.59) *p* = 0.76
Thoracic aorta	− 0.02 (− 0.49 to 0.45) *p* = 0.92	0.23 (− 0.34 to 0.68) *p* = 0.42	0.35 (− 0.22 to 0.74) *p* = 0.22

Data in bold indicate statistically detectable correlations

Statistical analysis was performed using the SAS v.9.4 (Institute Inc., Cary, NC, USA). A p-value < 0.05 was considered statistically detectable.

## Results

Patients were admitted to the hospital mainly because of pulmonary symptoms and persistent fever. COVID symptoms started 5–7 days before [^18^F]FDG PET/CT. Treatment was well tolerated without any side effects. Fever disappeared between day 3 and day 9 in all patients. All patients were discharged after a double negative nasopharyngeal of swab SARS-CoV-2.

Haematological data and quantitative [^18^F]FDG uptake in tissues at the basal time and at recovery are reported in Table 1.

Compared to control subjects, we found significantly lower lymphocyte count in patients at diagnosis vs. control subjects and higher monocyte count. Both parameters normalized at recovery. [^18^F]FDG uptake was higher in the lungs, spleen, terminal ileum, caecum, and right colon and normalized at recovery in all tissues but not in the right colon.

Half of the patients were moderately-lymphopenic at diagnosis, and half were severely lymphopenic (Table 2). In severely-lymphopenic patients, [^18^F]FDG uptake in the caecum and right colon was significantly higher than in moderately-lymphopenic patients. Furthermore, severely-lymphopenic patients, at diagnosis, had a significantly lower number of CD3 + CD4 + /CD45 + and CD16 + CD56 + /CD45 + cells, as compared to patients with moderate lymphopenia (Table 3).

**Fig. 2:**
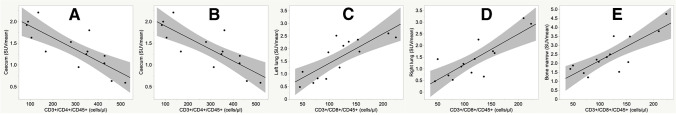
Graphical representation of the relation between CD4 + lymphocytes and [^18^F]FDG uptake in the caecum (**A**) and right colon wall (**B**) and between CD8 + cells and [^18^F]FDG uptake in lungs (**C** and **D**) and bone marrow (**E**)

Finally, by correlating SUV_mean_ values with immunological and serological parameters, we observed a direct correlation between [^18^F]FDG uptake in lungs or bone marrow with the absolute number of circulating CD3 + CD8 + /CD45 + cells and an inverse correlation between [^18^F]FDG uptake in the caecum and right colon with the absolute number of circulating CD3 + CD4 + /CD45 + cells (Table 4) thus, the less these cells circulate in blood, the more we find activity in the bowel wall (Fig. 2).

This finding suggests that CD4 + cells migrate in large bowel wall during active COVID-19, whereas CD8 + cells are mainly recruited in lungs and their presence in the blood is somehow related to bone marrow hyper-metabolism.

**Fig. 3:**
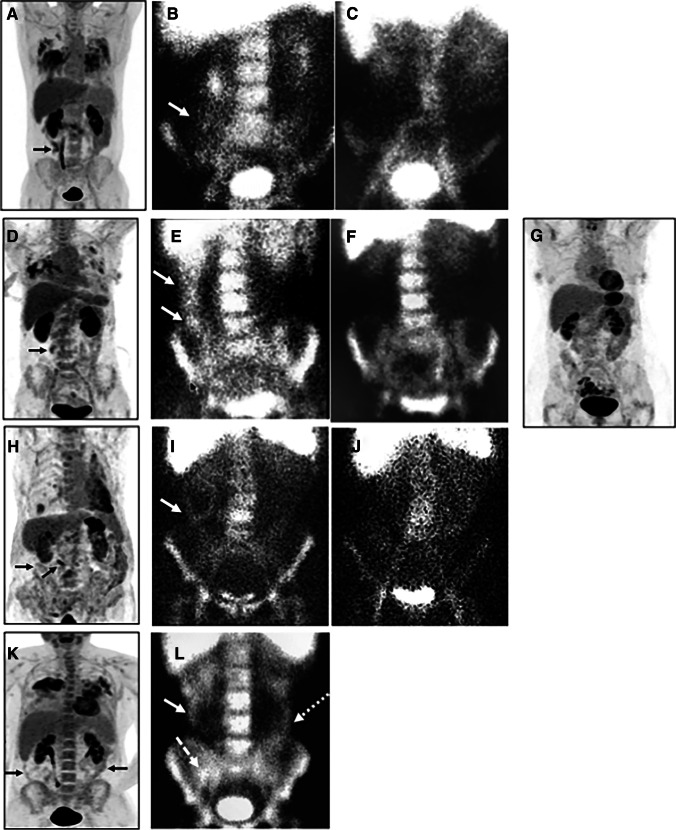
Anterior view of the abdomen was acquired 2 h after intravenous injection of radiolabelled leukocytes in 4 patients at the time of diagnosis of COVID-19 (**B**, **E**, **I**, **L**) and at recovery (**C**, **F**, **J**). All patients show the presence of radiolabelled autologous leukocytes in the right colon (solid arrows) and in the caecum (dashed arrow) and left colon (dotted arrow) in one case (**L**). Scans performed after recovery from COVID-19 (**C**, **F**, **J**) do not show any activity in the bowel. Bone marrow activity is also lower at recovery as compared to diagnosis. Maximum intensity projection (MIP) images after [^18^F]FDG of the same patients are also shown at the time of diagnosis (**A**, **D**, **H**, **K**) and after recovery (**G**). Activity in the lungs is clearly visible, as well as activity in the bowel (solid black arrows)

In the four patients that performed ^99m^Tc-WBC (Fig. 3), early images (when no bowel activity is detectable in normal subjects) showed the variable presence of radiolabelled leukocytes in the right colon, a sign of their migration in the bowel wall. One patient also clearly showed activity in the caecum and left colon. No detectable activity was found in the lungs. Remarkably, when patients recovered from COVID-19, they did not have any activity in the bowel, and the bone marrow also showed less uptake as compared to the scan at diagnosis.

## Discussion

We report, for the first time, the distribution of [^18^F]FDG in 18 patients with recent onset of COVID-19 and the relation with some haematological parameters. Indeed, we were motivated in understanding the severity and extent of inflammation in these patients. Interstitial pneumonia has been well described in several recent articles by CT scan [14–16] as well as the role of [^18^F]FDG PET/CT [17, 18]. We used [^18^F]FDG as a pan-inflammatory marker with a ROI-based quantitative analysis of SUVmean. SUVmean reflects the mean activity present in a given ROI, and it is more informative than the value of SUVmax (maximum uptake in a single pixel) or the target/background ratio (influenced by the possible variations of background activity in different patients), or SUVpeak (that is based on a selected region of high activity). Furthermore, we correlated [^18^F]FDG uptake in tissues with an absolute count of neutrophils, lymphocytes, monocytes, and several lymphocyte subsets. We found a significant inverse correlation between CD4 + cells and [^18^F]FDG uptake in the large bowel wall, which we interpreted as the migration of CD4 + cells in this organ. The [^18^F]FDG scans at recovery and the ^99m^Tc-WBC scans (both at diagnosis and at recovery), despite a small number of patients, strongly support our hypothesis. As control subjects, we chose age-matched patients with a normal biodistribution of FDG without any pathological uptake.

In a study carried out in China on 21 confirmed cases with COVID-19, levels of IL2, IL2 receptor (IL2R), IL6, IL10, and TNF-α were significantly higher in severe cases than in milder infections, and this was associated with a reduction of absolute numbers of total T-cells, CD4 + , and CD8 + in the majority of patients [19]. Other studies confirmed these findings. In another series of 41 COVID-19, lymphopenia and leukopenia were detected in 63 and 25% of patients, respectively [20].

Recently, Bay and coll analysed [^18^F]FDG distribution in convalescing COVID-19 patients and found high activity in the lungs, mediastinal nodes, liver, and spleen but not in the bowel wall [13]. Interestingly, they also found an inverse correlation between spleen uptake of [^18^F]FDG and lymphocyte count. Unfortunately, they did not investigate COVID-19 patients at diagnosis nor examined a possible relation with lymphocyte subsets, as we did. Furthermore, most of their patients were still lymphopenic, thus in a clinical phase between our newly diagnosed patients and recovered patients (all nonlymphopenic anymore).

The causes of lymphopenia, strictly related to cytokine storm, remain to be clarified. Therefore, understanding the underlying phenomena at the basis of lymphopenia, may be relevant for developing new therapeutic and prevention strategies.

We previously demonstrated that the severe lymphopenia caused by the administration of a humanised anti-CD3 monoclonal antibody (Visilizumab) is not due to T cell apoptosis but to rapid and massive margination of T cells in the spleen and large bowel [21]. Similarly, we hypothesize that T cells in COVID-19 may migrate from blood to peripheral tissues. Investigating the ileum and colon of COVID-19 patients is not feasible by colonoscopy due to the clinical conditions of these patients. Autopsy data are scarce, although lymphocytes in the bowel wall have been described [22, 23] as well as gastrointestinal symptoms [24], and SARS-CoV-2 has been detected in feces [25] and bowel epithelium [26] of COVID-19 patients. Despite none of our patients reporting gastrointestinal symptoms, a certain degree of large bowel inflammation was detected by [^18^F]FDG. This finding, together with the evidence of an inverse correlation between [^18^F]FDG uptake in the bowel and the absolute number of circulating CD4 + cells, was strongly supporting our hypothesis. Furthermore, we provide here evidence of the migration of radiolabelled autologous WBC into the large bowel wall in four out of four patients studied. Radiolabelled WBC is mainly neutrophils, with a small percentage of lymphocytes, and this could explain the high activity found in the liver and spleen and the faint uptake in the bowel. It is more difficult to explain why we did not observe any leukocyte migration in the lungs, despite the presence of pneumonia. A possibility, could be that in these patients (diagnosed on the same day of WBC scan) the pneumonia was not severe yet, with no WBC recruitment, but lymphopenia might have started with consequent migration of cells to the large bowel. When [^18^F]FDG PET/CT was performed (5–7 days later), pneumonia was indeed detectable. There is just one report in the literature of a WBC scan in a patient with COVID-19 in which lung uptake is detectable [27], but bowel activity was not investigated.

The finding of [^18^F]FDG activity in the bowel of patients with COVID-19 is not rare. A few reports have shown bowel activity [13, 28–30], but this has never been carefully investigated.

In a recently published paper [26], Trevelin and co. analysed post-mortem tissues from throughout the gastrointestinal tract (unfortunately excluding caecum and right colon) of patients who died from COVID-19 and found diffused intestinal inflammation with depleted Peyer’s patches (PP) and abundant macrophage infiltration in sub-mucosal tissue. Although these data were obtained in patients who died of severe COVID and mainly in the ileum, we cannot exclude that also, in patients with moderate COVID, there is a depletion of lymphocytes in PP with an inflammatory reaction and macrophage infiltration of the sub-mucosa also in the caecum and right colon. In this case, the high intestinal uptake of FDG may be due to macrophage infiltration rather than CD4 + cell infiltration. These data suggest the hypothesis that the inverse correlation between circulating CD4 + cells and colon uptake of FDG that we observed could be due to lymphopenia and unrelated bowel inflammation due to SARS-CoV-2 infection of bowel epithelium and macrophage infiltration. However, another post-mortem study [23] by Falasca and co. reported that COVID-19 patients have multi-organ diffuse lymphocytic infiltration, in contrast to what was reported by Trevelin et al. The presence of lymphocytes in the right colon and cecum, therefore, remains to be clarified and until then, both hypotheses should be taken into consideration. Indeed, our hypothesis is supported by the results of white blood cell scintigraphy.

Nevertheless, our study has some limitations, mainly due to the difficulty to organise scans and dealing with COVID-19 positive patients at diagnosis. Many patients were not enrolled because of severe COVID-19 and under oxygen therapy or because images were acquired too many days after clinical diagnosis. A contrast agent for CT scan was not used, but slice thickness of 3.75 mm, minimizes mismatches between CT and PET scan, and PET scan started from the pelvic region immediately after CT acquisition. Finally, the caecum and ascending colon do not usually have such a high peristaltic activity in fasting conditions. WBC scan is also not easy to perform because of the blood manipulation involved. Furthermore, our patients were certainly not infected with the Omicron variant of the SARS-CoV-2 virus, which is most frequently observed now. We do not have data about the virus variant in patients, but given the date of recruitment, it is likely that all patients had the original variant of the SARS-CoV-2 virus. Lymphopenia, however, is being found in all patients with COVID-19, and the understanding of the pathophysiology of this phenomenon may have important consequences in the development of new therapeutic strategies.

The recent availability of radiopharmaceuticalS that specifically target CD4 + [31] or CD25 + cells [32] could provide more information on the trafficking and distribution of these cell subsets in COVID-19 patients.

## Conclusions

In conclusion, as compared to normal subjects, patients with newly diagnosed COVID-19 syndrome and lymphopenia showed glucose hyper-metabolism in the lungs, spleen, ileum, caecum, and right colon wall, which can be interpreted as an inflammatory reaction in these organs. After recovery from COVID-19, these organs tend to normalize, but the right colon remains more inflamed than controls. The inverse correlation between circulating CD4 + cells and [^18^F]FDG uptake in the bowel wall suggests that CD4 + cells may migrate in this organ, contributing to inflammation. This finding is also indirectly supported by the evidence of bowel uptake of ^99m^Tc-WBC at the time of diagnosis.

The direct confirmation of our findings may arise from future studies aiming at investigating the trafficking in vivo of radiolabelled CD4 + cells. In the meantime, our study opens a new hypothesis on the pathogenesis of lymphopenia in patients with COVID-19 and may stimulate the search for new therapeutic strategies.

## Supplementary Information

Below is the link to the electronic supplementary material.

Supplementary file1 (DOCX 15 KB)
