# Supplementary material for: Lymphopenia in patients affected by SARS-CoV-2 infection is caused by margination of lymphocytes in large bowel: an [18F]FDG PET/CT study
Source: Eur J Nucl Med Mol Imaging. 2022 Apr 29;49(10):3419–29. doi: 10.1007/s00259-022-05801-0 (PMC9050483; doi:10.1007/s00259-022-05801-0)
Supplement: Supplementary file 1 — Supplementary file1 (DOCX 15 KB) [file 259_2022_5801_MOESM1_ESM.docx]

**Supplementary table**

| **Parameter** | **Covid-19 patients** | **Control subjects** | **p** |
| --- | --- | --- | --- |
| Gender (Female/Male (%)) | 4 (22.2)/14 (77.8) | 12 (66.6)/6 (33.3) | **0.007** |
| Age* (mean±SD; (95%CI)) | 60.29±9.47; (55.9 to 64.7) | 58.71±8.92; (54.6 to 62.8) | 0.61 |
| Reason for performing FDG-PET (n (%)) |  |  | **<0.0001** |
| Breast cancer | 0 (0.0) | 2 (11.1) |  |
| Cervical cancer | 0 (0.0) | 1 (5.6) |  |
| Covid-19 | 18 (100.0) | 0 (0.0) |  |
| External auditory canal cancer | 0 (0.0) | 1 (5.6) |  |
| Lung cancer | 0 (0.0) | 1 (5.6) |  |
| Non-Hodgkin's lymphoma | 0 (0.0) | 2 (11.1) |  |
| Solitary pulmonary nodule | 0 (0.0) | 9 (50.0) |  |
| Thyroid cancer | 0 (0.0) | 1 (5.6) |  |
| Melanoma | 0 (0.0) | 1 (5.6) |  |
| Co-morbidities |  |  | 0.99 |
| Dyslipidemia | 2 (11.1) | 1 (5.6) |  |
| Cardiovascular disease | 0 (0.0) | 2 (11.1) |  |
| Gastritis/Reflux | 1 (5.6) | 2 (11.1) |  |
| Multinodular goiter | 0 (0.0) | 1 (5..6) |  |
